# Establishment of a fish model to study gas-bubble lesions

**DOI:** 10.1038/s41598-022-10539-8

**Published:** 2022-04-21

**Authors:** Alicia Velázquez-Wallraf, Antonio Fernández, María José Caballero, Marina Arregui, Óscar González Díaz, Mónica B. Betancor, Yara Bernaldo de Quirós

**Affiliations:** 1 Veterinary Histology and Pathology, Atlantic Center for Cetacean Research, University Institute of Animal Health and Food Safety (IUSA), Veterinary School, University of Las Palmas de Gran Canaria (ULPGC), Canary Islands, Spain; 2 Physical and Chemical Instrumental Center for the Development of Applied Research Technology and Scientific Estate, Institute for Environmental Studies and Natural Resources (I-UNAT), University of Las Palmas de Gran Canaria (ULPGC), Las Palmas, Spain; 3 Institute of Aquaculture, Faculty of Natural Sciences, University of Stirling, Stirling, UK; 4 Department of Integrative Physiology, University of Colorado Boulder, Boulder, CO USA

**Keywords:** Vascular diseases, Experimental models of disease

## Abstract

Decompression sickness (DCS) is a clinical syndrome caused by the formation of systemic intravascular and extravascular gas bubbles. The presence of these bubbles in blood vessels is known as gas embolism. DCS has been described in humans and animals such as sea turtles and cetaceans. To delve deeper into DCS, experimental models in terrestrial mammals subjected to compression/decompression in a hyperbaric chamber have been used. Fish can suffer from gas bubble disease (GBD), characterized by the formation of intravascular and extravascular systemic gas bubbles, similarly to that observed in DCS. Given these similarities and the fact that fish develop this disease naturally in supersaturated water, they could be used as an alternative experimental model for the study of the pathophysiological aspect of gas bubbles. The objective of this study was to obtain a reproducible model for GBD in fish by an engineering system and a complete pathological study, validating this model for the study of the physiopathology of gas related lesions in DCS. A massive and severe GBD was achieved by exposing the fish for 18 h to TDG values of 108–109%, characterized by the presence of severe hemorrhages and the visualization of massive quantities of macroscopic and microscopic gas bubbles, systemically distributed, circulating through different large vessels of experimental fish. These pathological findings were the same as those described in small mammals for the study of explosive DCS by hyperbaric chamber, validating the translational usefulness of this first fish model to study the gas-bubbles lesions associated to DCS from a pathological standpoint.

## Introduction

Decompression sickness (DCS) is a clinical syndrome described mainly in professional and recreational scuba divers, presenting symptoms such as muscle and joint pain, difficult breathing, weakness, dizziness, numbness, skin rashes, and neurological symptoms among others^1^. DCS was first described in 1878 by Bert^2^, reporting these symptoms in caisson workers returning to the surface after finishing their shifts underwater. This disease is caused by the formation of systemic intravascular and extravascular gas bubbles when the sum of the partial pressures of dissolved gases in the tissues exceeds the atmospheric pressure^3^. The presence of gas bubbles in blood vessels is known as gas embolism and can lead to mechanical and biochemical alterations.

DCS has also been described in wild animals such as sea turtles^4^ and cetaceans^5–12^, presumably caused by the alteration of their diving profile and diving response with regards to a stressor^4,7^. In most cases, these alterations were associated with anthropogenic activities such as the use of high-intensity mid-frequency sonar in military naval maneuvers coincident in time and space with mass strandings^5–8^ or bycatch^4^, but it has also been described in stressing large prey interactions^12^. A severe form of DCS has been described in these species, namely explosive decompression sickness characterized by a lethal gas embolism, which in humans rarely occurs except in cases of severe diving accidents^13^.

To delve deeper into DCS, experimental models in terrestrial mammals subjected to compression/decompression in a hyperbaric chamber have been used. In some of these models, mainly developed in rats and rabbits, compressions were performed for a variable period (6–8 absolute atmospheres, 40–90 min) followed by rapid decompression (3–5 min), aiming to induce severe DCS^14–19^. These models have allowed comparison with marine mammals affected by DCS, showing very similar pathology findings^20^.

Fish can suffer from gas bubble disease (GBD), characterized by the formation of intravascular and extravascular systemic gas bubbles, similarly to gas bubbles observed in DCS. GBD has been extensively studied, mainly in large dam areas where the discharge of significant amounts of water from one dam level to another entrains and dissolves atmospheric air causing an increase in total dissolved gases (TDG) in the water^21^. Clinical signs have been observed in live fish inhabiting these supersaturated waters, such as loss of buoyancy and erratic swimming, along with pathological signs such as gas bubbles in eyes and fins^22^. Fish experimental studies on GBD have mainly focused on the survival of the different fish species and life stages at different TDG values^21–34^.

Several authors have described the usefulness of an in vivo model of GBD to study gas embolism related to DCS, from the perspective of the formation of intravascular gas bubbles, their growth and subsequent endothelial pathophysiology, that are common to both diseases^35–37^. Given the similarities between GBD and DCS and the fact that fish develop this disease naturally, they could be used as an alternative experimental model following the replacement principle in accordance with European laboratory animal regulations (Directive 2010/63/EU of the European Parliament). Hence, the main objective of the present study was to obtain a reproducible model for massive GBD in fish. In order to achieve this aim, an engineering system was implemented to produce and maintain over time high concentrations of TDG, and the clinical diagnosis of GBD was confirmed with a complete pathological study validating, for the first time, this fish model to study the gas bubbles associated lesions similar to DCS from a pathological standpoint.

## Results

### Generation of supersaturated water with an open aquarium

**Figure 1:**
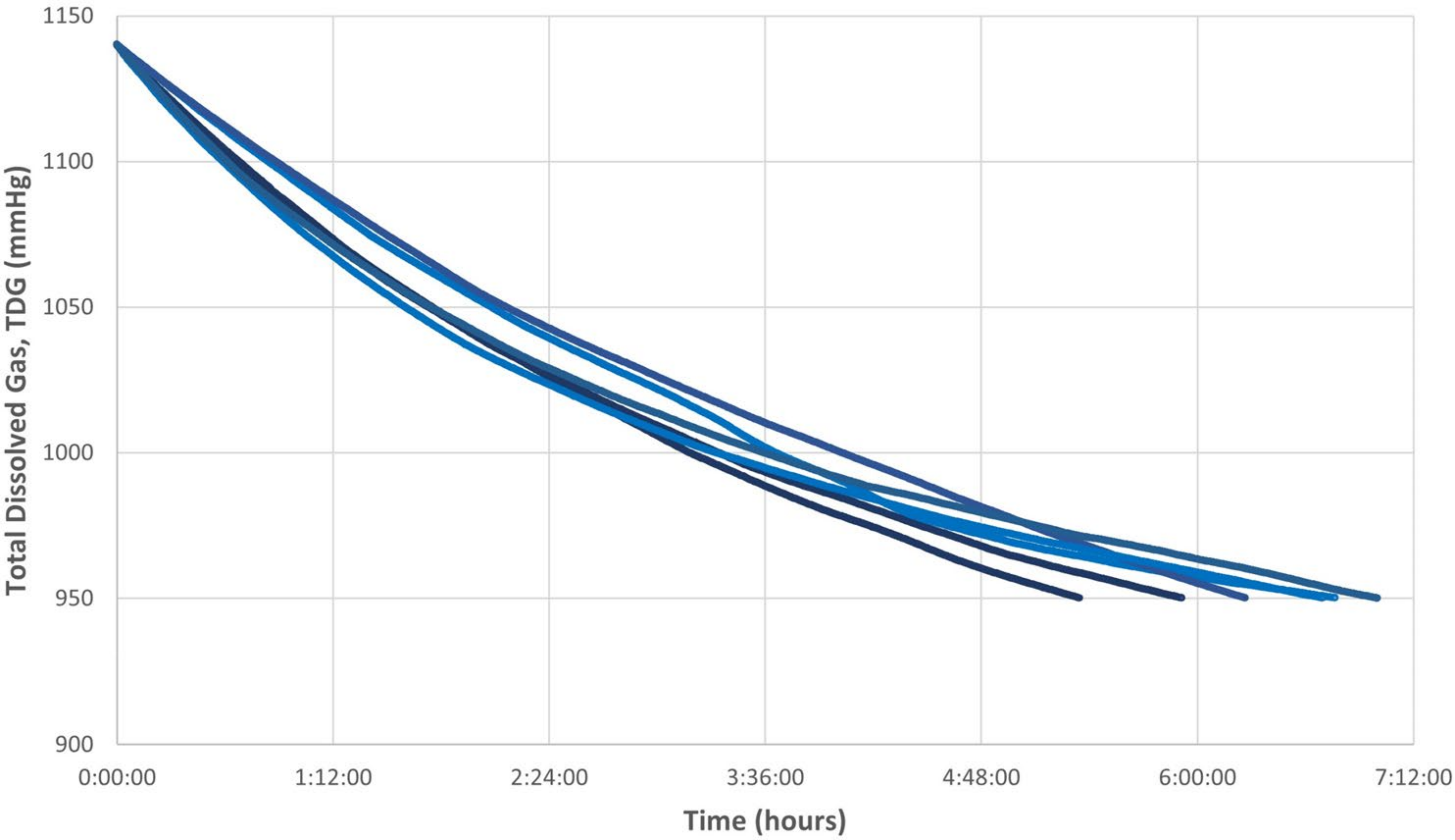
Graphical representation of the evolution of supersaturated water in an open aquarium without recirculation based on experiments performed from 1140 mmHg (150% of TDG). Each line corresponds to six different experiments with a similar pattern of TDG decrease in all of them.

An engineering system was designed (see “Methods”) to produce supersaturated water with high percentages of TDG following the existing literature^38,39^. Once the desired TDG was achieved, the supersaturated water was transferred to an open aquarium, where it remained stagnant. The results showed a decrease of TDG with time when using the open aquarium and without recirculating the water. This decrease was observed from a value of 150–125% in approximately 6 h (Fig. 1).

### Generation of supersaturated water with a pressurized aquarium

**Figure 2:**
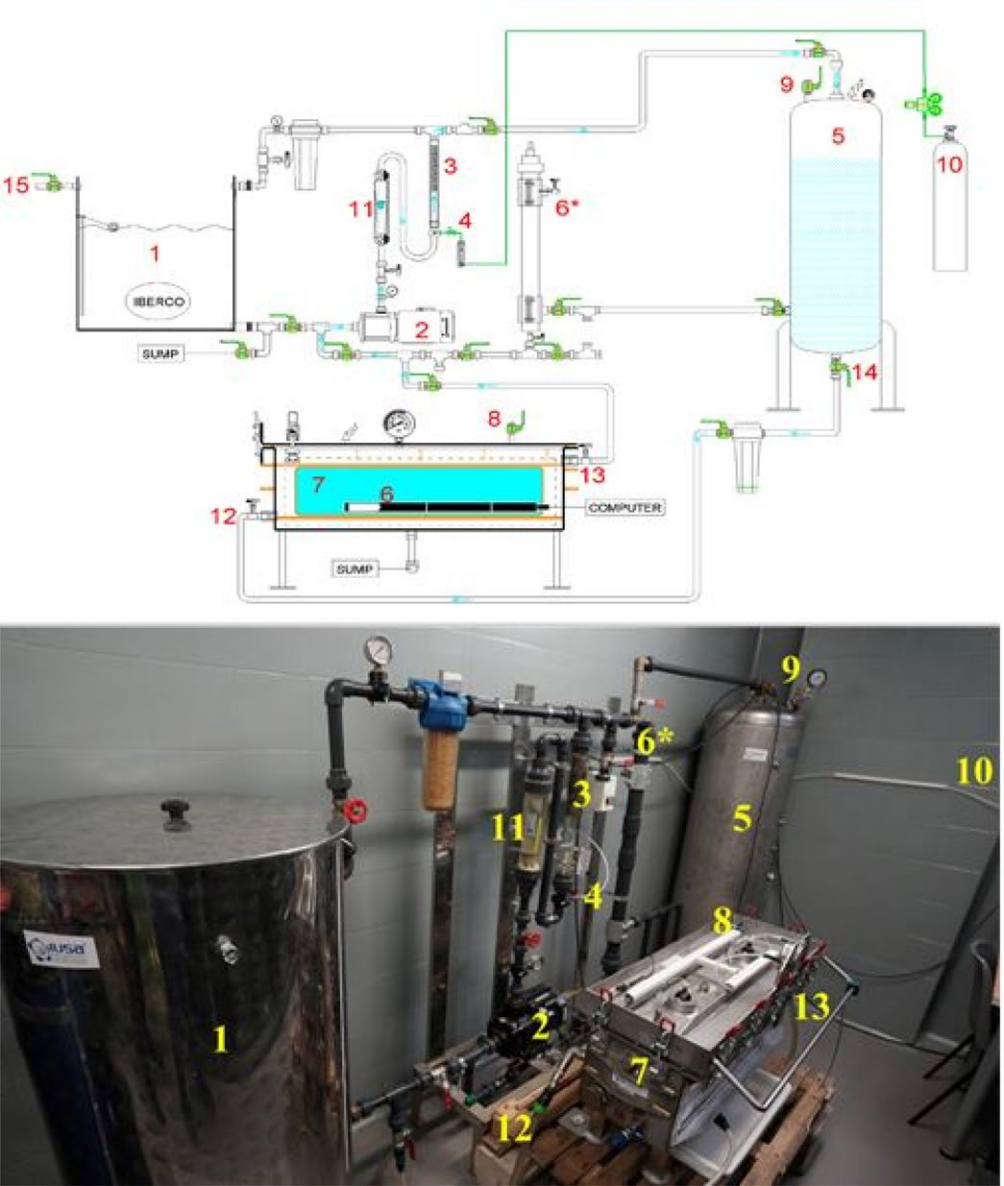
Supersaturated water production system components. (1) Open vessel. (2) Motor pump. (3) Dissolution tube. (4) Synthetic air injection valve. (5) Pressurized tank. (6) TDG and temperature sensor. (6*) TDG and temperature sensor inside the open circuit. (7) Pressurized aquarium. (8) Pressurized aquarium vent valve. (9) Pressurized tank vent valve. (10) Synthetic air bottle. (11) Flow meter. (12) Pressurized aquarium inlet valve. (13) Pressurized aquarium outlet valve. (14) Pressurized tank outlet valve. (15) Running water inlet.

**Figure 3:**
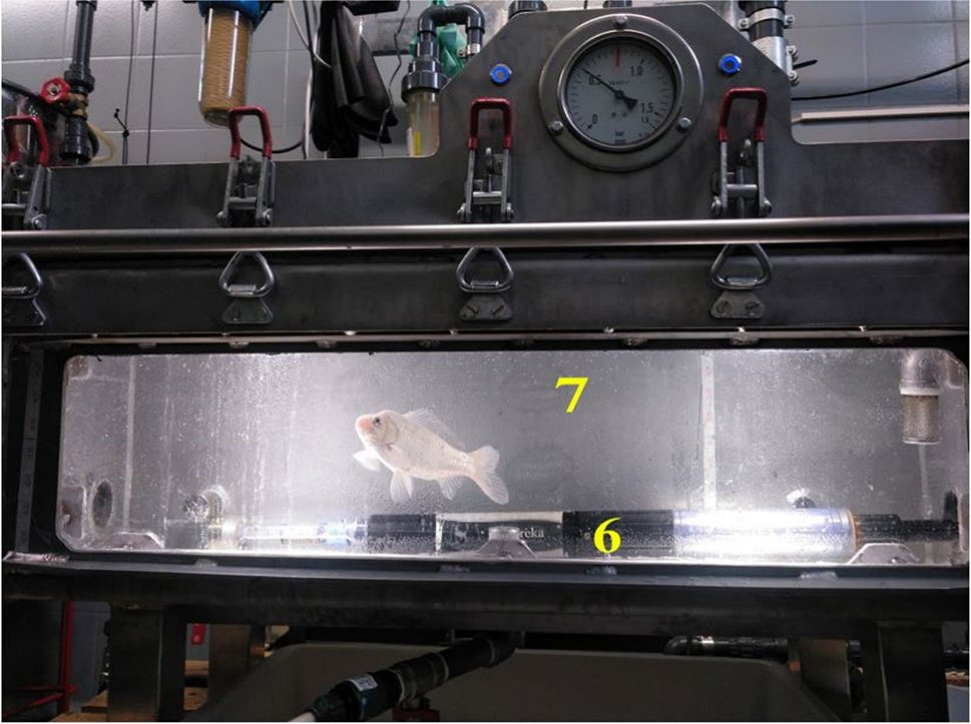
Detail of pressurized aquarium during a fish experiment. TDG and temperature sensor (6). Pressurized aquarium (7).

The significant decrease in TDG forced to modify the initial circuit by designing and introducing, for the first time, a pressurized aquarium (Fig. 2), creating a closed circuit with constant recirculation (Fig. 3). This new design was able to produce TDG water of around 107% and 120% successfully and to maintain it stable over time.

**Figure 4:**
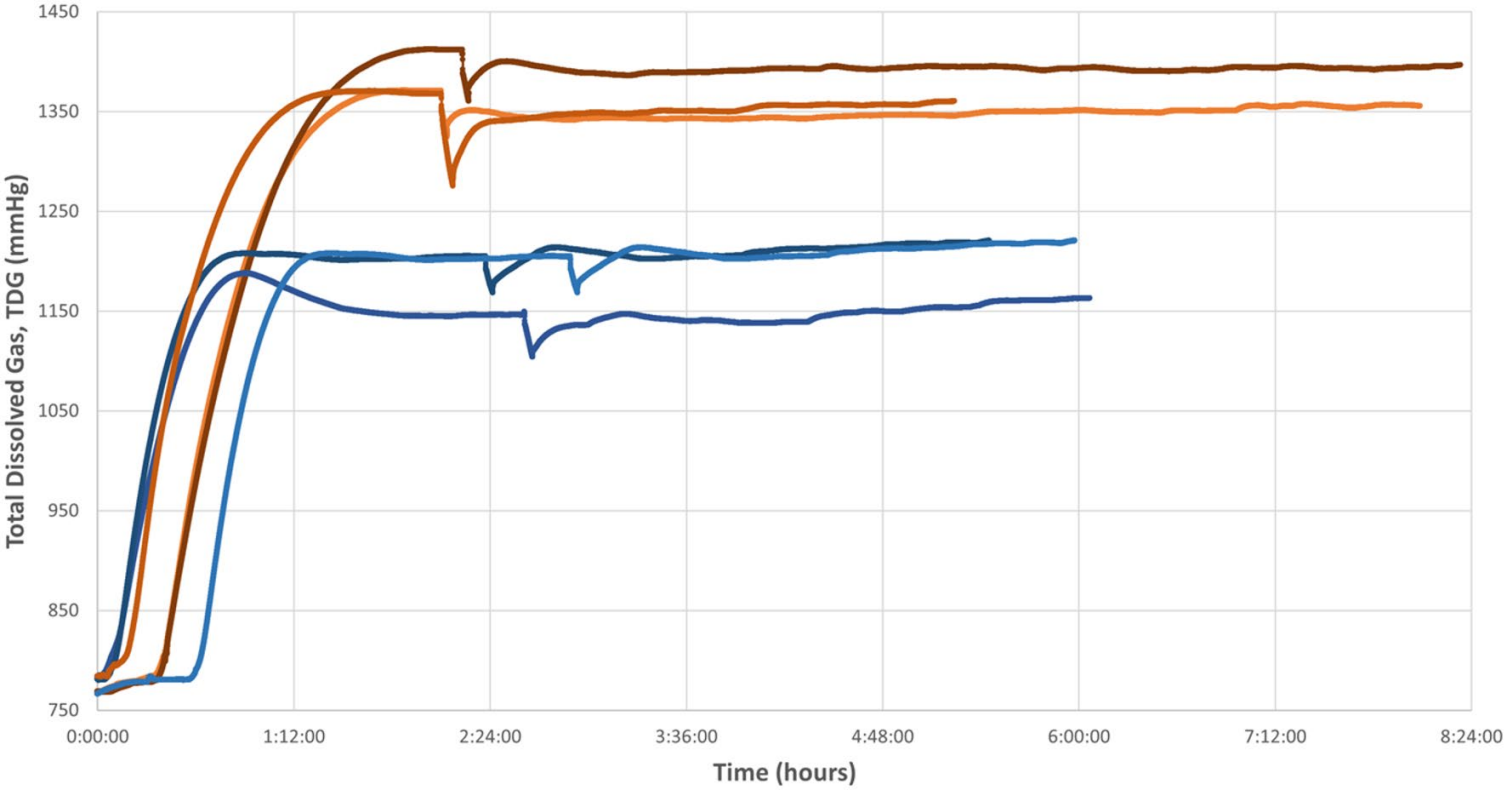
TDG plots represent the pilot tests without fish. The orange lines represent the group in which a high TDG value (120%) was reached, while the blue lines represent the experiments with a TDG of approximately 101%. The peaks observed in the different tests represent the simulation of fish introduction.

The time at which it was simulated to introduce a fish into the pressurized aquarium (see “Methods”) had no effect on TDG values, since this simulation caused a slight decrease due to depressurization followed by fast recovery of TDG. Figure 4 shows the simulation maneuver of fish introduction through the descending peak and rapid recovery to stable values, behaving similarly in the different trials performed with different TDG values and times.

**Figure 5:**
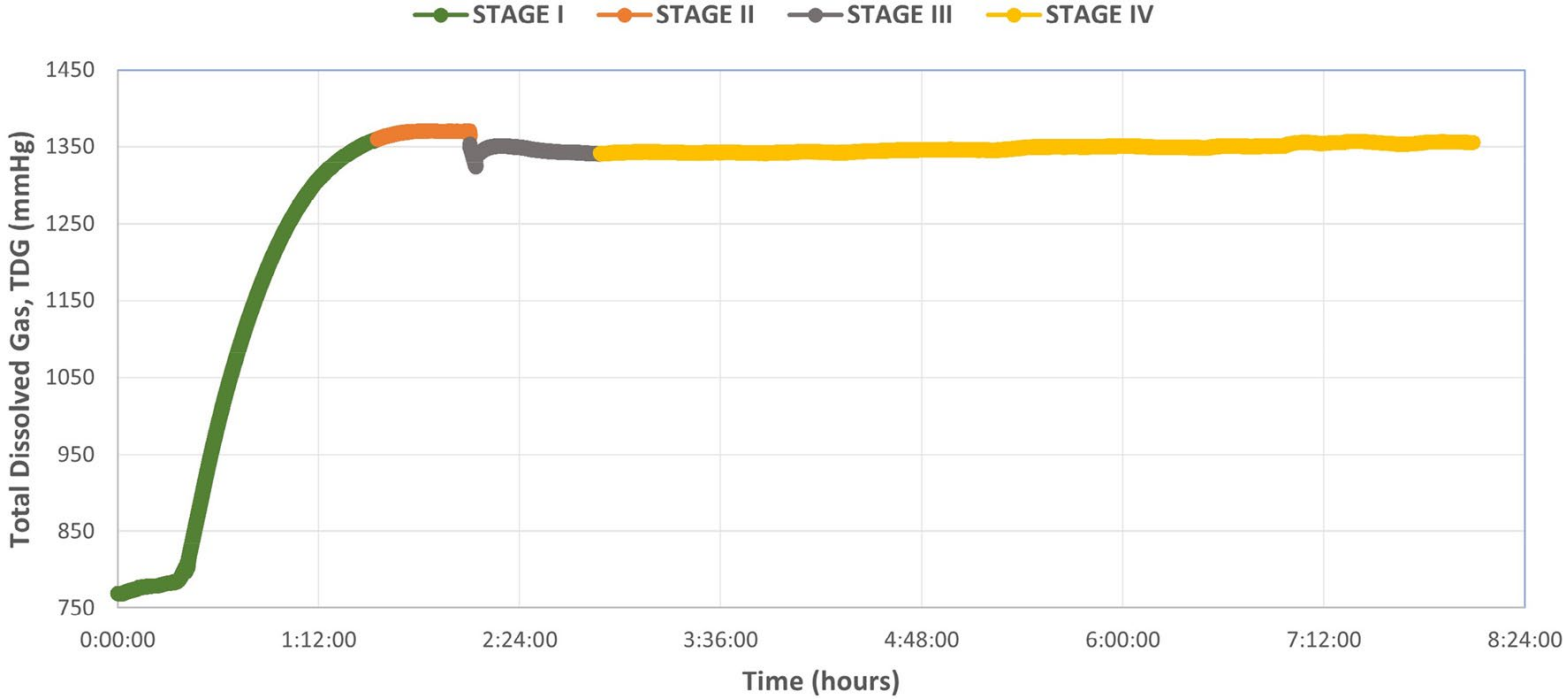
Diagram of the 4 phases into which the water supersaturation procedure can be divided. Phase I (green) represents the increase of TDG in the water due to the addition of compressed air from a cylinder. Phase II (orange): stabilization or plateau phase of the TDG value. Phase III (gray): simulation of the introduction of a fish into the pressurized aquarium representing the loss of TDG when depressurizing the aquarium and the rapid recovery of the value. Phase IV (yellow): stabilization of TDG post-simulation, which remains constant over time.

Therefore, a model graph of the TDG behavior is established (Fig. 5) that shows the global overview of the tests performed without fish. The TDG values over time graphs can be divided into different stages: stage I (green) in which the supersaturation of the flowing water occurs, stage II (orange) in which the maximum TDG value for a given experiment is reached and is maintained stable (plateau), stage III (gray) where TDG values dropped briefly and abruptly (reverse peak) with a quick recovery to a TDG value closed to stage II, reflecting the isolation and opening of the pressurized aquarium to simulate the introduction of the fish and the subsequent recirculation of supersaturated water once the aquarium was closed again, and stage IV (yellow), where TDG values are stable again (Fig. 5).

The pilot test results showed that the maximum TDG was reached (stage II) after 90 min, although 30–60 more minutes were necessary to confirm that the TDG was stable, at which time the fish may be introduced.

### Pilot tests: fish exposure

**Figure 6:**
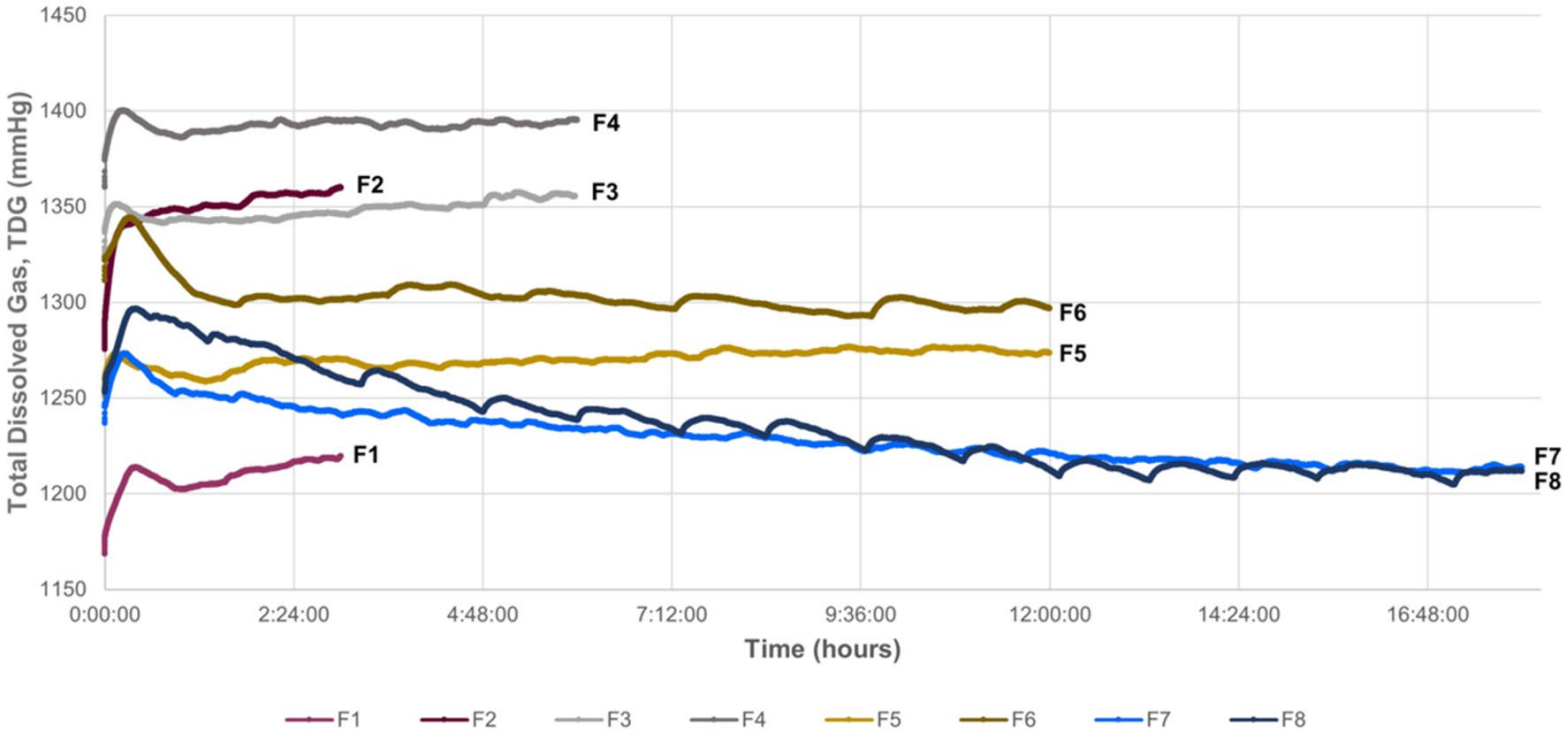
Representation of the evolution of TDG values from the introduction of the fish to the end of the exposure time for each experimental fish. F1-F2 correspond to the 3 h group, F3–F4 to the 6 h group, F5–F6 to the 12 h group, and F7–F8 to the 18 h model group.

Fish were exposed to TDG values between 107 and 120%. TDG was maintained stable in all tests (Fig. 6). The 3 h, 6 h, and 12 h groups presented GBD, although it was not massive, regardless of exposure to very high TDG values, so the 18 h group was sequentially resorted to. The 18 h group was initially planned for a time exposure of 24 h, following the same reasoning of doubling the hours from the previous group treatment, but both fish reached the humane endpoint at 18 h, showing signs of loss of buoyancy and lethargy. TDG average values for the 18 h group was of 108% and 109%.

### Clinical signs

The clinical signs of the 18 h-fish group are described below (details of all groups are provided in the Supplementary Material). Thus, both fish from the 18 h group presented the same clinical signs when introduced into the pressurized aquarium. They immediately presented intense agitation, denoted by swimming back and forth, being three times faster than control fish. In addition, they showed intense opercular beat frequency, two times faster than control fish. Between 5 and 10 min after the experiment started, fish were paralyzed at the bottom of the pressurized aquarium. From then on, they were less steady and tried to swim, slowly recovering their regular swimming pattern and speed and their standard opercular beat frequency. Small gas bubbles were visible in the lateral and anal fins after 30 min of exposure, becoming visually larger in between 1 and 2 h of exposure. Between 6 and 12 h of the experiment, many gas bubbles in all fins were recorded. In the last interval (12–18 h), the fish presented some loss of scales. At 17 h of exposure, the fish returned to the bottom of the pressurized aquarium, showed erratic movements, loss of buoyancy, and rigidity of the pectoral fins with very severe hemorrhages, congestion, and gas bubbles, as well as in the rest of the fins. The fish were euthanized at this point, following the pre-established humane endpoint.

### Macroscopic findings

**Figure 7:**
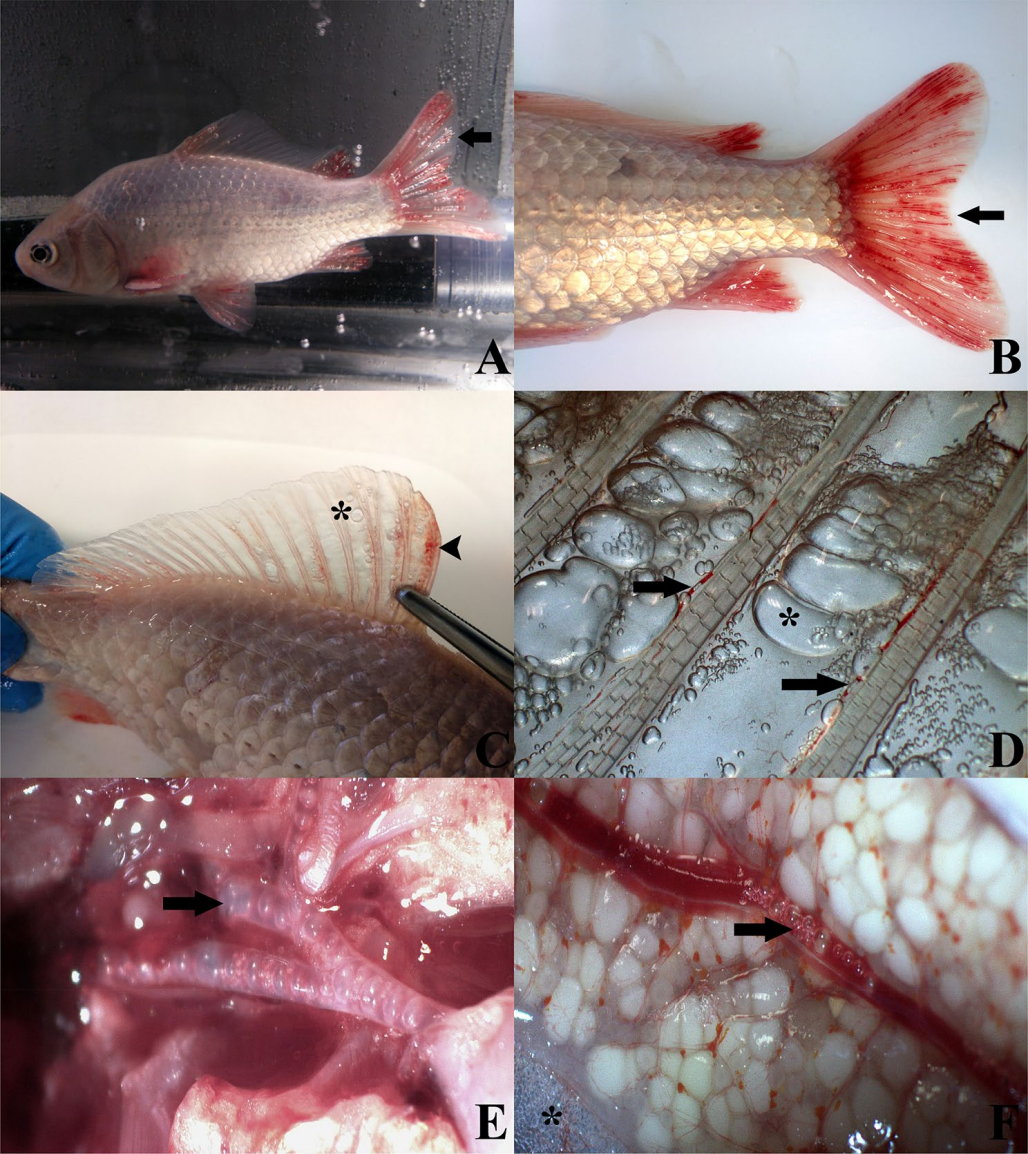
Macroscopic appearance of the 18-h fish group. (**A**) Fish alive inside the pressurized aquarium in the last hours of the experiment. Emphysema, congestion, and hemorrhages are observed in all fins. (**B**) External examination of the fish after euthanasia. Presence of the similar lesions observed in vivo. (**C**) Dorsal fin with the presence of emphysema (star) and multifocal hemorrhages (arrowhead). (**D**) Detail of dorsal fin with gas bubbles in blood vessels (arrows) and emphysema (star). (**E**) Ventral aorta at its exit from the bulbus arteriosus filled with gas. (**F**) Gas bubbles circulating in the gonadal vessel and presence of emphysema of the adjacent adipose tissue.

The macroscopic findings of the 18 h-fish group are described below (details of all groups are provided in the Supplementary Material). All fins presented severe emphysema, multifocal hemorrhagic areas, and gas bubbles within the congestive vessels, either in vivo (Fig. 7A) and post-euthanasia (Fig. 7B–D). Gas bubbles were also observed within the vessels of the medial side of the opercula. Additionally, the fish presented loss of scales, mainly around the base of the tail, where the skin was crepitant to the touch denoting mild subcutaneous emphysema. The gills presented severe congestion.

Internal examination of the organs revealed the presence of large amounts of gas bubbles in the heart, ventral aorta (Fig. 7E), posterior cardinal veins, gonads veins (Fig. 7F), swim bladder vasculature, and in the intercostal vessels. In addition, severe generalized organ congestion, denoted mainly in liver, kidney, and spleen, and moderate emphysema of visceral fat were also observed.

### Microscopic findings

**Figure 8:**
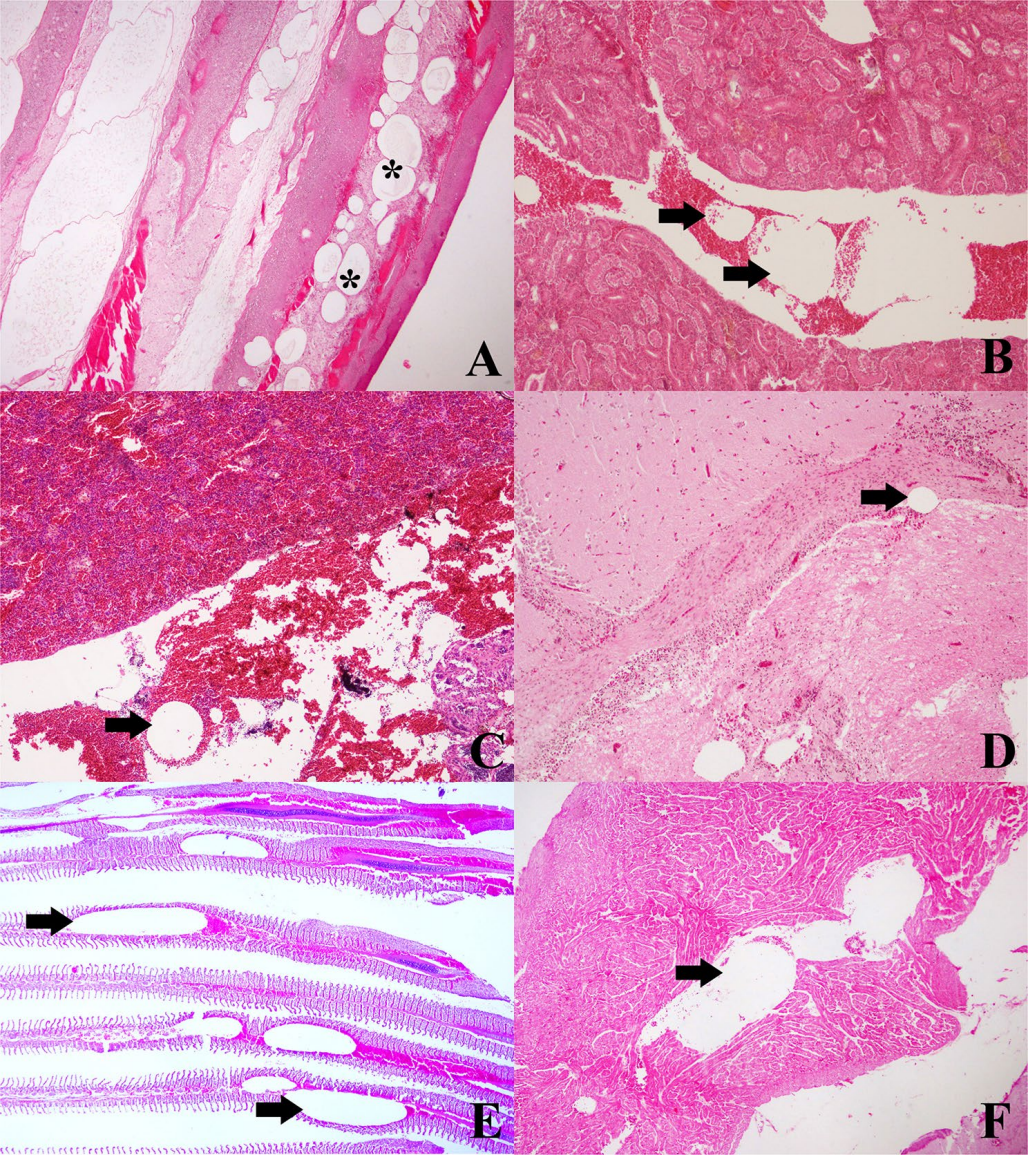
Microscopic findings of the 18-h fish group stained routinely with hematoxylin–eosin. (**A**) Fins displays the presence of emphysema (stars) and congestion. ×4. (**B**) Posterior kidney with large, unstained, circular to oval structures between blood components (arrows). ×10. (**C**) Gas bubble-like in the spleen (arrow), along with congestion. ×10. (**D**) Congestion and dilatation of blood vessels in the brain (arrow). ×10. (**E**) Blood vessels of primary gill lamellae with large caliber bubble-like gas, circumscribed by blood cells (arrows). ×4. (**F**) Ventricle cavity of the heart with presence of displaced blood around an oval, unstained structure compatible with gas bubble (arrow). ×4.

The microscopic findings of the 18 h-fish group are described below (details of all groups are provided in the Supplementary Material). Large non-staining dilatations compatible with emphysema and severe congestion were observed in fins (Fig. 8A), along with moderate multifocal hemorrhages and some intravascular bubble-like round empty spaces among blood cells. Gills presented severe congestive areas with large gas bubbles circulating within capillaries associated with primary lamellae. Severe congestion was also observed in spleen and posterior kidney, with the latter showing some hemorrhagic areas among the renal tubules. Rete mirabile of swim bladder, liver, ocular structures, and central nervous system showed moderate congestive appearance. The same circular structures were observed among the blood displacing the erythrocytes inside vascular structures of posterior kidney (Fig. 8B), spleen (Fig. 8C), liver, digestive tract, eyes, central nervous system (Fig. 8D), gills (Fig. 8E), and between blood of heart’s ventricle (Fig. 8F).

## Discussion

A massive GBD in fish with the presence of severe gas-bubble lesions similar to that seen in explosive DCS in other species. (e.g., humans, cetaceans, rabbits, rats)^4–19^ was achieved by exposing the fish for 18 h to TDG values of 108–109%, validating a new experimental model from the point of view of the study of gas bubble pathophysiology, alternative to rabbits and rats following the 3Rs replacement principle.

The Directive 2010/63/EU of the European Parliament states that in the case that the use of live animals is necessary, an animal species with a lower capacity to feel pain, suffering, distress, or lasting harm that can be extrapolated to the target species should be used. Following these reasons, the development of this work and the use of fish as a model organism for DCS has a great value on the development of new approaches that allow the implementation of the 3R’s principles.

Unlike rabbits and rats, fish can develop GBD naturally when large quantities of water fall from one dam level to another, entraining atmospheric gas, causing an increase in TDG. D'Aoust and Smith^35^ were the first authors to describe GBD in fish as a useful in vivo model to study DCS. From the point of view of the development of gas bubbles, first intravascular and well perfused tissues, their growth and the consequences triggered by their presence, Speare^36^ also considered GBD as an interesting model to deepen in the pathophysiological aspects of gas embolism in DCS.

Most previous studies on the GBD in fish focused on the tolerance and survival of different species and life stages to specific TDG values^21,28,39^. The present study differs from these previous manuscripts in the goal and approach since the aim was to reproduce a massive GBD as a model to study systemic massive gas bubbles related to explosive DCS.

The first step in order to achieve a model of massive GBD was to be able to produce water saturated with high levels of TDG and to maintain those levels along the duration of the experiment. Most previous experimental studies described stable TDG values while recirculating water with an open tank^39^. However, we could not recirculate the water in these conditions due to the differences in pressure between the open aquarium and the rest of the circuit, preventing the correct operation of the motor pump and causing more water outflow than inflow, leading to overflowing of the aquarium. Hence, the supersaturated water was transferred from the tank to the open aquarium and remained steady to observe how the TDG values behaved. In this case, the loss of TDG was almost immediately causing a rapid exponential decrease, as shown in Fig. 1. For this reason, a pressurized aquarium was added to the design, enabling the same pressure throughout the circuit and allowing constant recirculation of water. As a result, TDG values remained constant in these conditions during the study period (Fig. 4).

Once the stable parameters were achieved, the next step was to perform the experiments by introducing the fish. Although most experiments on GBD in supersaturated water have been conducted with fish of the family *Cyprinida*e, none have been conducted with *Carassius auratus*. The choice of this species was based on the fact that carp are fish that acclimatize very well to new environments, are economically accessible, and are easily marketed.

The pathology of GBD, in non-experimental studies, is described in both living and dead fish as gas bubbles and hemorrhages in fins^28,32,34,40^, as well as gill congestion and hemorrhages, along with exophthalmia^31,41^. In addition, microscopic lesions such as gas bubbles in blood vessels, hemorrhages, and congestion in different tissues, attributed to GBD, have been described^42,43^. In the first test performed (3 h with TDG 107–119%) fish showed signs of GBD but at a mild level, with presence of few gas bubbles in fins and vascular locations, together with mild hemorrhages and organic congestion. The aim of establishing a comparative model of gas-bubbles lesions from DCS lead to the development of a massive GBD model where the presence of extravascular and intravascular gas bubbles is severe and massive, throughout most of the vascular system. By sequentially repeating the tests and doubling the exposure time we reproduced this massive GBD model, after 18 h of exposure to TDG values of 108–109%. This model was characterized by the presence of severe hemorrhages, congestion, and gas bubbles in the fins as well as the visualization of circulating macroscopic gas bubbles by different large vessels (Fig. 7), coupled with microscopic confirmation of massive quantities of gas bubbles systematically distributed, hemorrhages, and multi-organ congestion (Fig. 8). Overall, pathological findings were the same as those described in small mammals for the study of gas-bubbles lesions in explosive DCS by hyperbaric chamber^14,16,18,20^, validating the translational usefulness of this model from a pathological standpoint.

As described by Marking^44^, gas excess in the water is rapidly transmitted to the bloodstream of the fish as the osmotic pressures between the two sides of the gill membrane tend to equalize and both blood and water have similar saturation points. This might explain why gas bubbles form in fish with low supersaturations, in comparison with other species.

The main limitation of this experimental model in fish is from the point of view of raising the questions of how and when intravascular and extravascular gas bubbles are formed. Therefore, it could not be applied to studies focused on the origin and formation of these gas bubbles in human DCS, but it could be considered for the study of the consequences of these gas bubbles.

In conclusion, a massive GBD with severe intravascular and extravascular gas bubbles similar to gas-bubbles lesions related to DCS was reproduced when goldfish were exposed during 18 h to TDG values between 108 and 109% in a pressurized aquarium. The clinical diagnose was confirmed by means of a pathological study. This represents the first establishment of a fish model as an alternative model to rodents and other mammals for the study of gas bubbles produced in DCS in humans and it could be a useful model to deepen in the pathophysiology of gas bubbles in tissues.

## Methods

### Generation of supersaturated water with an open aquarium

Following the scheme of Bouck et al.^38^ and taking into account that the dissolution of gas in water depends on pressure and temperature, in order to obtain supersaturated water at a constant temperature, it is necessary to exert pressure. Thus, these authors, by microdispersing water in an aeration tank, forced the gas to dissolve. Therefore, with this scheme as baseline^38^, a dissolution tube was designed, filled with porous materials of decreasing diameters, which broke the gas bubbles from the air injected. Supersaturated water was obtained forcing, with constant recirculation, the dissolution of gas and, therefore, increasing the TDG of the water.

The engineering circuit (IBERCO S.L., Spain) was composed of several structures shown in Fig. 2: (1) an open vessel which was filled with water from the tap, (2) a motor pump (Grundfos, Model: CM 10-1 ARAE-AVBE CAAN) to circulate water within the circuit, (3) a dissolution tube with (4) air injector, (5) a pressurized tank (max. 3 ATA; 250 L) to increase the pressure and favor the dissolution of gas into water and to store it, (6*) a TDG sensor (Manta + Trimeter probe, Eureka water probes, USA) placed within the circuit to control for TDG values. Once the desired TDG value was reached in the pressurized tank, the water was transferred to the open aquarium, and the TDG sensor was moved into the aquarium to ensure TDG exposure along the duration of the experiments.

### Generation of supersaturated water with a pressurized aquarium

The circuit was modified by adding a (7) pressurized aquarium (Fig. 3) (max 0.5 bar; 70 L) (IBERCO S.L., Spain). To produce supersaturated water (Fig. 2), first (1) the open vessel was filled with tap water at ambient pressure (the vent valves from (8) the pressurized aquarium and (9) tank were opened) with the help of (2) the motor pump. Then, both vent valves were closed, and (10) pressurized synthetic atmospheric air (Premier-X50S 200.0B) was injected (4). The amount of synthetic air injected was regulated using a flow meter connected to the circuit. When the maximum pressure of the circuit was reached (0.5 bar), no more air was injected, and the water was continuously recirculated (3000 L/h) from the dissolution tube to the pressurized tank and vice versa for several minutes to favor the dissolution of the gas remaining in the head space of the pressurized tank until a dynamic equilibrium between the gas and the liquid phase within the circuit was reached (determined by a constant reading of TDG values) and to ensure a homogenous TDG water mixture.

### Pilot tests: parameter settings without fish

To adjust the parameters of the experimental model, different tests without fish were initially carried out following the supersaturated water production scheme explained above. In these pilot tests, the amount of synthetic air injected and recirculating time was varied, with the main objective of achieving a stable TDG value of more than 101% during the entire experiment. To test how high TDG values the system could produce and maintain stability over time, two different TDG ranges of around 101% and 120% TDG were tested. These values were reached by setting the outlet pressure of the synthetic air bottle at 3 bar, with a flow rate of 10–20 L/min for 4–6 min.

Since in the fish exposure experiments, it is necessary to open the pressurized aquarium in order to introduce the fish, causing an inevitable pressure loss in the circuit, the effect of this pressure drop in the TDG was analyzed by simulating the introduction of the fish during the pilot tests. Once the TDG was stabilized around the desired value, the recirculation of the water was stopped, and the aquarium was depressurized and opened, simulating the introduction of a fish. Then, the aquarium was closed, and the water was recirculated again through the circuit. We tested for different times at which introducing the fish.

### Pilot tests: fish exposure

10 adult goldfish (*Carassius auratus*) of 112.8 ± 11.74 g and 16.95 ± 0.60 cm were purchased from a certified fish distributor in Las Palmas de Gran Canaria (Spain) and acclimatized during 4 weeks in tanks at the experimental animal facility (EGC00616436) of the University of Las Palmas de Gran Canaria (Spain). The fish were kept under a natural photoperiod of 12:12 h light:dark cycle. Water chemistry parameters were assessed every 2 days using a colorimetric test kit (nitrate, 10 mg/dm^3^; nitrite, 0 mg/dm^3^; pH, 6.8; total hardness, 80–300 mg/dm^3^; chlorine, 0 mg/dm^3^). Dissolved oxygen (> 6.0 mg/ dm^3^) and temperature (23–25 °C) were also measured. Fish were fed a commercial pellet (Tetra Goldfish) diet two times a day.

Fish experiments (n = 8) were performed sequentially by increasing time exposure and TDG values to determine the parameters triggering a massive GBD in adult goldfish, which was confirmed through pathological studies. The experiments started with 3 h of exposure. If massive production of gas bubbles were not observed, then the time was increased (3 h (h), 6 h, 12 h, and 18 h). TDG values were established based on the results of previous non-fish experiments so that these were in the range of 107–120%. These values reflect this range of amplitude since TDG tends to stabilize as exposure time increases. A humane endpoint (18 h) was established when the fish showed loss of buoyancy, erratic swimming, and lethargy. Two control animals were placed in the pressurized aquarium without recirculation of water for 18 h.

### Clinical signs

Clinical monitoring of the fish was performed throughout the exposure time, with the observation of fish behavior as well as swimming and opercular beat frequency. In addition, the presence of possible external lesions such as the appearance of gas bubbles and hemorrhages in fins and loss of scales or subcutaneous emphysema were monitored. Thus, the intense opercular beat frequency value was established when the value was 2–4 times faster than normal based on control fish (± 2 openings/s). In the same way, intense agitation was determined when fish swim 2–3 times faster than regular swimming based on control fish (± 10 s swimming from one side of the aquarium to the other).

### Macroscopic evaluation

Fish were euthanized with 2-phenoxyethanol (0.6 ml/L). Necropsy was immediately performed by a thorough external examination with an especial focus on describing lesions on fins, eyes, integument, and gills. Similarly, the coelomic cavity was dissected carefully from the anus to the ventral area of the operculum via the midline to expose the organs and visualize the blood vessels. A stereo microscope was used for the visualization of gas bubbles.

### Microscopic evaluation

Samples of gill, fins, heart, swim bladder, digestive tract, liver, spleen, anterior and posterior kidney, gonads, muscle, eyes, and central nervous system were collected in 10% formalin for subsequent histopathological study. The formalin-fixed tissues were dehydrated, cleared, and embedded in paraffin, and sectioned at 4 μm. The obtained samples were routinely stained with hematoxylin and eosin (H&E) and microscopically observed by two pathologists.

### Approval for animal experiments

Experimental procedures were approved by the ethical committee of the University of Las Palmas de Gran Canaria and authorized by the competent authority of the Canary Islands Government (CEEA-ULPGC 4-2018R1). All the trials were designed and performed to result in the death of as few animals as possible and reduce the duration and intensity of suffering according to the relevant guidelines and regulations (Directive 2010/63/EU). Authors complied with the ARRIVE guidelines.

### Supplementary Information

Supplementary Information.

## Data Availability

All data generated during and/or analyzed during the current study are available from the corresponding author on reasonable request.
