# Author Correction: Establishment of a fish model to study gas-bubble lesions

**DOI:** 10.1038/s41598-024-52889-5

**Published:** 2024-01-30

**Authors:** Alicia Velázquez-Wallraf, Antonio Fernández, María José Caballero, Marina Arregui, Óscar González Díaz, Mónica B. Betancor, Yara Bernaldo de Quirós

**Affiliations:** 1 Veterinary Histology and Pathology, Atlantic Center for Cetacean Research, University Institute of Animal Health and Food Safety (IUSA), Veterinary School, University of Las Palmas de Gran Canaria (ULPGC), Canary Islands, Spain; 2 Physical and Chemical Instrumental Center for the Development of Applied Research Technology and Scientific Estate, Institute for Environmental Studies and Natural Resources (I-UNAT), University of Las Palmas de Gran Canaria (ULPGC), Las Palmas, Spain; 3 Institute of Aquaculture, Faculty of Natural Sciences, University of Stirling, Stirling, UK; 4 Department of Integrative Physiology, University of Colorado Boulder, Boulder, CO USA

Correction to: *Scientific Reports* 10.1038/s41598-022-10539-8, published online 21 April 2022

The original version of this Article contained errors.

In the original version of this Article, the saturation % of total dissolved gases (TDG) in the pressurized aquarium values was incorrectly calculated for an ambient pressure of 1 ATA, instead of 1.5 ATA.

As a result, in the Abstract section, where

“A massive and severe GBD was achieved by exposing the fish for 18 h to TDG values of 162–163%”

now reads

“A massive and severe GBD was achieved by exposing the fish for 18 h to TDG values of 108–109%”

In addition, in the Results section, where

“This new design was able to produce TDG water of around 160% and 180% successfully and to maintain it stable over time.”

now reads

“This new design was able to produce TDG water of around 107% and 120% successfully and to maintain it stable over time.”

where

“Fish were exposed to TDG values between 160 and 180%. TDG was maintained stable in all tests (Fig. 6). The 3 h, 6 h, and 12 h groups presented GBD, although it was not massive, regardless of exposure to very high TDG values, so the 18 h group was sequentially resorted to. The 18 h group was initially planned for a time exposure of 24 h, following the same reasoning of doubling the hours from the previous group treatment, but both fish reached the humane endpoint at 18 h, showing signs of loss of buoyancy and lethargy. TDG average values for the 18 h group was of 162% and 163%.”

now reads

“Fish were exposed to TDG values between 107 and 120%. TDG was maintained stable in all tests (Fig. 6). The 3 h, 6 h, and 12 h groups presented GBD, although it was not massive, regardless of exposure to very high TDG values, so the 18 h group was sequentially resorted to. The 18 h group was initially planned for a time exposure of 24 h, following the same reasoning of doubling the hours from the previous group treatment, but both fish reached the humane endpoint at 18 h, showing signs of loss of buoyancy and lethargy. TDG average values for the 18 h group were 108% and 109%.”

Furthermore, in the Discussion section, where

“A massive GBD in fish with the presence of severe gas-bubble lesions similar to that seen in explosive DCS in other species. (e.g., humans, cetaceans, rabbits, rats) 4–19 was achieved by exposing the fish for 18 h to TDG values of 162–163%,”

now reads

“A massive GBD in fish with the presence of severe gas-bubble lesions similar to that seen in explosive DCS in other species. (e.g., humans, cetaceans, rabbits, rats)4–19 was achieved by exposing the fish for 18 h to TDG values of 108–109%,”

where

“In the first test performed (3 h with TDG 160–178%) fish showed signs of GBD but at a mild level, with presence of few gas bubbles in fins and vascular locations, together with mild hemorrhages and organic congestion.”

now reads

“In the first test performed (3 h with TDG 107–119%) fish showed signs of GBD but at a mild level, with presence of few gas bubbles in fins and vascular locations, together with mild hemorrhages and organic congestion.”

where

“By sequentially repeating the tests and doubling the exposure time we reproduced this massive GBD model, after 18 h of exposure to TDG values of 162–163%.”

now reads

“By sequentially repeating the tests and doubling the exposure time we reproduced this massive GBD model, after 18 h of exposure to TDG values of 108–109%.”

and where

“In conclusion, a massive GBD with severe intravascular and extravascular gas bubbles similar to gas-bubbles lesions related to DCS was reproduced when goldfish were exposed during 18 h to TDG values between 162 and 163% in a pressurized aquarium.”

now reads

“In conclusion, a massive GBD with severe intravascular and extravascular gas bubbles similar to gas-bubbles lesions related to DCS was reproduced when goldfish were exposed during 18 h to TDG values between 108 and 109% in a pressurized aquarium.”

Moreover, under the Methods section, where

“In these pilot tests, the amount of synthetic air injected, and recirculating time was varied, with the main objective of achieving a stable TDG value of more than 150% during the entire experiment. To test how high TDG values the system could produce and maintain stability over time, two different TDG ranges of around 150% and 180% TDG were tested.”

now reads

“In these pilot tests, the amount of synthetic air injected, and recirculating time was varied, with the main objective of achieving a stable TDG value of more than 101% during the entire experiment. To test how high TDG values the system could produce and maintain stability over time, two different TDG ranges of around 101% and 120% TDG were tested.”

and where

“TDG values were established based on the results of previous non-fish experiments so that these were in the range of 160–180%.”

now reads

“TDG values were established based on the results of previous non-fish experiments so that these were in the range of 107–120%.”

Finally, in the caption of Figure 4, where

“TDG plots represent the pilot tests without fish. The orange lines represent the group in which a high TDG value (180%) was reached, while the blue lines represent the experiments with a TDG of approximately 150%. The peaks observed in the different tests represent the simulation of fish introduction.”

now reads

“TDG plots represent the pilot tests without fish. The orange lines represent the group in which a high TDG value (120%) was reached, while the blue lines represent the experiments with a TDG of approximately 101%. The peaks observed in the different tests represent the simulation of fish introduction.

Absolute values in mm of Hg shown in the text and graphs are correct.

The original version of this Article has been corrected.

