# Supplementary material for: Establishment of a fish model to study gas-bubble lesions
Source: Sci Rep. 2022 Apr 21;12:6592. doi: 10.1038/s41598-022-10539-8 (PMC9023494; doi:10.1038/s41598-022-10539-8)
Supplement: Supplementary file 1 — Supplementary Information. [file 41598_2022_10539_MOESM1_ESM.pdf]

Supplementary table 1. Detail of clinical signs during the experiment observed in each animal at different time intervals.

CLINICAL SIGNS	Group 3 hours		Group 6 hours		Group 12 hours		Group 18 hours	
	**Fish 1**	**Fish 2**	**Fish 1**	**Fish 2**	**Fish 1**	**Fish 2**	**Fish 1**	**Fish 2**
**0-10 minutes**	- Intense agitation*<br>- Intense opercular beat frequency**	- Intense agitation*<br>- Intense opercular beat frequency **	- Intense agitation*<br>- Intense opercular beat frequency **	- Intense opercular beat frequency **<br>- Paralyzed at the bottom of the aquarium	- Intense agitation*	- Intense agitation*<br>- Intense opercular beat frequency **	- Intense agitation*<br>- Intense opercular beat frequency **	- Intense opercular beat frequency **<br>- Paralyzed at the bottom of the aquarium
**10-30 minutes**	-	-	-	- Remains at the bottom of the aquarium	- Paralyzed at the bottom of the aquarium	- Paralyzed at the bottom of the aquarium	- Paralyzed at the bottom of the aquarium	- Kept at the bottom of the aquarium
**30 minutes-1 hour**	- Less agitated and normal opercular beat frequency	- Less agitated and normal opercular beat frequency	- Less agitated and normal opercular beat frequency	- Less paralyzed, sometimes swimming.<br>- Normal opercular beat frequency	- Less paralyzed, sometimes swimming	- Less paralyzed, sometimes swimming<br>- Normal opercular beat frequency	- Less paralyzed, sometimes swimming<br>- Normal opercular beat frequency<br>- Doubtful bubbles in anal and lateral fins	- Less paralyzed, sometimes swimming<br>- Normal opercular beat frequency
**1-1:30 hour**	- Regains normal swimming	- Regains normal swimming<br>- Doubtful bubbles in caudal fin.	- Mixed behavior: sometimes swimming and sometimes paralyzed on the bottom<br>- First evidence of bubbles in pectoral and dorsal fins	- Regains normal swimming	- Mixed behavior: sometimes swimming and sometimes paralyzed on the bottom.	- Regains normal swimming<br>- Doubtful bubbles in all fins.	- Regains normal swimming<br>- Evidence of bubbles in all fins	- Regains normal swimming<br>- Doubtful bubbles seen in all fins
**1:30-2 hours**	- Paralyzed on the bottom.<br>- Evidence of bubbles in dorsal, anal fins and some bubbles in caudal fin	- Evidence of bubbles in caudal fin, some bubbles in the rest of fins.	- Spasmodic movement of body	-	-	-	-	- Clear bubbles in all fins
**2-3 hours**	- Spasmodic movements of body<br>- Lethargic	-	- Fewer spasmodic movements	-	-	- Evidence of bubbles in all fins<br>- Spasmodic movement of body	-	- Spasmodic movement of body
**3-6 hours**			- Regains normal swimming	-	-	-	- Mixed behavior: sometimes swimming and sometimes paralyzed on the bottom	-
**6-12 hours**					- Many bubbles in all fins and some hemorrhages<br>- Loss of some scales	- Many bubbles in all fins	-	- Many bubbles in all fins
**12-18 hours**							- Lethargic and sometimes swimming with erratic movements<br>- Many bubbles, congestion and hemorrhages in fins<br>- Subcutaneous emphysema with loss of scales	- Lethargic and sometimes swimming with erratic movements<br>- Many bubbles, congestion and hemorrhages in fins<br>- Subcutaneous emphysema with loss of scales

*Intense agitation: 3 times faster than normal agitation based on control fish (±10 seconds swimming from one side of the aquarium to the other).
**Intense opercular beat frequency: 2 times faster than normal opercular beat frequency based on control fish (±2 openings/second).

Supplementary table 2. Details of the macroscopic findings observed in the organs analyzed in each animal.

MACROSCOPIC FINDINGS	Group 3 hours		Group 6 hours		Group 12 hours		Group 18 hours	
	**Fish 1**	**Fish 2**	**Fish 1**	**Fish 2**	**Fish 1**	**Fish 2**	**Fish 1**	**Fish 2**
**FINS**	- Caudal fin: mild congestion and emphysema<br>- Rest: mild emphysema	- Caudal fin: mild emphysema, focal hemorrhage and mild congestion<br>- Rest: mild emphysema	- Caudal and dorsal fin: mild emphysema and congestion<br>- Rest: mild emphysema	- Ventral, pectoral and anal fins: moderate emphysema<br>- Rest: mild emphysema	- All fins: moderate emphysema, with the focal hemorrhage and moderate congestion	- All fins: moderate emphysema.<br>- Caudal fins: presence of multifocal hemorrhages	- All fins: severe emphysema with hemorrhagic areas and bubbles observed within the vessels	- Pectoral fins: severe congestion, multifocal hemorrhages and severe emphysema<br>- Bubbles inside the vessels of these fins<br>- Caudal and dorsal fins: less affected but with similar lesions
**INTEGUMENT**	-	-	-	-	- Loss of some scales	-	- Loss of scales and mild subcutaneous emphysema	- Mild subcutaneous emphysema<br>- Loss of some scales
**GILLS**	-	-	-	-	- Moderate congestion with presence of some bubbles	- Moderate congestion<br>- Hemorrhage in right opercular medial aspect	- Severe congestion	- Severe congestion<br>- In the medial wall of both opercula, bubbles inside the vessels are observed
**EYES**	-	- Mild exophthalmia	- Mild exophthalmia	-	-	- Mild left exophthalmia and some right exophthalmia	-	-
**COELOMIC CAVITY**	- Mild generalized congestion	- Mild generalized congestion	- Mild generalized congestion	- Mild generalized congestion	- Moderate generalized congestion	- Moderate generalized congestion	- Severe generalized congestion	- Severe generalized congestion
**ADIPOSE TISSUE**	-	-	- Mild emphysema	- Mild emphysema	- Mild emphysema and, in some areas, moderate emphysema	- Moderate emphysema	- Moderate emphysema	- Moderate emphysema
**INTERCOSTAL VESSELS**	- Some bubbles	- Some bubbles at confluence with posterior kidney	- One bubble near the caudal pole of the posterior kidney	- Some circulating bubbles	- Some bubbles	- Some bubbles	- Several circulating bubbles	- Several bubbles in the caudal area
**POSTERIOR CARDINAL VEINS**	- Some bubbles	- Some circulating bubbles	- Some bubbles	- Some bubbles	- Some bubbles in its course	- Several bubbles circulating	- Several bubbles seen	- Several bubbles
**PORTAL VEINS**	-	- One bubble	-	- Not evaluated	- Not evaluated	-	- Some bubbles circulating	- Not evaluated
**SWIMBLADDER VEIN**	-	-	-	-	- Some bubbles	- Some bubbles	- Many bubbles between blood	- Many bubbles
**GONADAL VEINS**	-	-	-	-	-	-	- Some bubbles	- Some bubbles
**HEART**	-	-	-	-	- Ventral aorta and bulbus arteriosus with some bubbles	- Ventral aorta and bulbus arteriosus with some bubbles	- Several bubbles in ventral aorta transiting to the arterial bulb	- Ventral aorta and bulbus arteriosus full of bubbles
**POSTERIOR KIDNEY**	- Mild congestion	- Mild congestion	-	- Moderate congestion<br>- Hematoma in the caudal area	- Moderate congestion	- Moderate congestion	- Severe congestion	- Severe congestion

Supplementary table 3. Detail of the microscopic findings observed in the analyzed organs of each animal.

MICROSCOPIC FINDINGS	Group 3 hours		Group 6 hours		Group 12 hours		Group 18 hours	
	**Fish 1**	**Fish 2**	**Fish 1**	**Fish 2**	**Fish 1**	**Fish 2**	**Fish 1**	**Fish 2**
**FINS**	- Mild congestion<br>- Moderate emphysema	- Mild congestion<br>- Moderate emphysema	- Mild congestion<br>- Moderate emphysema<br>- Mild focal hemorrhages	- Mild congestion with few bubbles<br>- Moderate emphysema<br>- Mild focal hemorrhages	- Moderate congestion with some bubbles<br>- Severe emphysema<br>- Moderate focal hemorrhages	- Moderate congestion<br>- Severe emphysema<br>- Moderate multifocal hemorrhages	- Severe congestion of vessels with some bubbles inside<br>- Severe emphysema<br>- Moderate multifocal hemorrhages	- Severe congestion with some bubbles inside the vessels<br>- Severe emphysema<br>- Moderate multifocal hemorrhages
**GILLS**	- Mild congestion	- Mild congestion	- Moderate congestion<br>- Hyperplasia of secondary lamellae	- Moderate congestion	- Moderate congestion	- Moderate congestion	- Severe congestion with hyperplasia and fusion of secondary lamellae<br>- Vessels with large caliber bubbles	- Severe congestion<br>- Many vessels with large bubbles inside, scattered among the blood
**LIVER**	- Mild congestion	- Mild congestion	- Mild congestion<br>- Focal hemorrhage	- Mild congestion	- Moderate congestion	- Moderate congestion	- Moderate congestion<br>- One bubble	- Moderate congestion
**SPLEEN**	- Moderate congestion	- Moderate congestion	- Moderate congestion	- Moderate congestion<br>- Some focal bubbles	- Severe congestion	- Severe congestion<br>- Some focal bubbles	- Severe congestion<br>- Some focal bubbles	- Severe congestion<br>- Some bubbles
**DIGESTIVE TRACT**	- Mild congestion	- Mild congestion	- Mild congestion	- Mild congestion	- Mild congestion	- Mild congestion<br>- Areas of dilatation between muscular and serous layer of the intestine	- Moderate congestion<br>- Areas of dilatation between muscular and serous layer of the intestine	- Moderate congestion<br>- Some bubbles in capillaries
**GONADS**	-	-	- Mild congestion	-	- Mild congestion	- Mild congestion	- Mild congestion<br>- Focal areas of mild hemorrhages	- Mild congestion<br>- Some areas of mild hemorrhages
**SWIM BLADDER**	-	-	-	-	- Mild congestion of rete mirabile	- Mild congestion<br>- Tympanized	- Moderate congestion<br>- Tympanized	- Moderate congestion<br>- Tympanized
**POSTERIOR KIDNEY**	- Moderate congestion<br>- Some bubbles inside vascular structures<br>- Mild focal hemorrhages	- Moderate congestion<br>- Mild focal hemorrhages<br>- Some bubbles inside vascular structures	- Moderate congestion<br>- Some bubbles inside vascular structures<br>- Focal hemorrhages	- Moderate congestion<br>- Some bubbles inside vascular structures	- Moderate congestion<br>- Some bubbles inside vascular structures	- Moderate-severe congestion<br>- Some bubbles inside vascular structures<br>- Severe hemorrhages in some areas	- Severe congestion<br>- Many large caliber bubbles inside vascular structures	- Severe congestion<br>- Many coalescent bubbles inside blood vessels<br>- Moderate multifocal hemorrhages
**HEART**	- Some areas of mild hypereosinophilia	- Mild focal areas of hypereosinophilia	- Mild hypereosinophilia in some areas	- Focal mild vacuolization.	- Mild hypereosinophilia in some fibers	- Mild vacuolization in some fibers<br>- Few bubbles between blood of the cavity	- Mild hypereosinophilia of some areas<br>- Large caliber bubble between blood	- Mild hypereosinophilia and vacuolization in some fibers<br>- Some bubbles among the blood
**CENTRAL NERVOUS SYSTEM**	- Mild congestion	- Mild congestion	- Mild congestion	- Mild congestion	- Moderate congestion	- Moderate congestion	- Moderate congestion<br>- Some dilated vascular structures	- Moderate congestion<br>- Presence of a bubble in a cerebral capillary
**EYES**	-	-	-	-	- Mild congestion	- Mild congestion	- Moderate congestion<br>- One bubble	- Moderate congestion<br>- Few bubbles
**MUSCLE**	-	-	-	-	-	- Mild hypereosinophilia in some areas	- Mild hypereosinophilia in some areas	- Mild hypereosinophilia in some areas
